# A Candidate-Gene Association Study for Berry Colour and Anthocyanin Content in *Vitis vinifera* L.

**DOI:** 10.1371/journal.pone.0046021

**Published:** 2012-09-28

**Authors:** Silvana Cardoso, Winston Lau, José Eiras Dias, Pedro Fevereiro, Nikolas Maniatis

**Affiliations:** 1 Laboratory of Plant Cell Biotechnology, Instituto de Tecnologia Química e Biológica, Oeiras, Portugal; 2 Department of Genetics, Evolution and Environment, University College London, London, United Kingdom; 3 Instituto Nacional de Investigação Agrária, Instituto Nacional de Recursos Biológicos, Dois Portos, Portugal; Cairo University, Egypt

## Abstract

Anthocyanin content is a trait of major interest in *Vitis vinifera* L. These compounds affect grape and wine quality, and have beneficial effects on human health. A candidate-gene approach was used to identify genetic variants associated with anthocyanin content in grape berries. A total of 445 polymorphisms were identified in 5 genes encoding transcription factors and 10 genes involved in either the biosynthetic pathway or transport of anthocyanins. A total of 124 SNPs were selected to examine association with a wide range of phenotypes based on RP-HPLC analysis and visual characterization. The phenotypes were total skin anthocyanin (TSA) concentration but also specific types of anthocyanins and relative abundance. The visual assessment was based on OIV (*Organisation Internationale de la Vigne et du Vin*) descriptors for berry and skin colour. The genes encoding the transcription factors *MYB11*, *MYBCC* and *MYC_B_* were significantly associated with TSA concentration. *UFGT* and *MRP* were associated with several different types of anthocyanins. Skin and pulp colour were associated with nine genes (*MYB11*, *MYBCC*, *MYC_B_*, *UFGT*, *MRP*, *DFR*, *LDOX*, *CHI* and *GST*). Pulp colour was associated with a similar group of 11 genes (*MYB11*, *MYBCC*, *MYC_B_*, *MYC_A_*, *UFGT*, *MRP*, *GST*, *DFR*, *LDOX*, *CHI* and *CHS_A_*). Statistical interactions were observed between SNPs within the transcription factors *MYB11*, *MYBCC* and *MYC_B_*. SNPs within *LDOX* interacted with *MYB11* and *MYC_B_*, while SNPs within *CHI* interacted with *MYB11* only. Together, these findings suggest the involvement of these genes in anthocyanin content and on the regulation of anthocyanin biosynthesis. This work forms a benchmark for replication and functional studies.

## Introduction

Anthocyanins are natural pigments which accumulate especially in fruits and flowers [1]. These compounds, which are part of the larger group of flavonoids [2], [3], play a very important role in wine and grape industry. This is because they confer colour and contribute to other organoleptic characteristics [4]. They can also be used as food colourants and as antioxidants with several benefits for human health [5].

The biosynthetic pathway of anthocyanins is well characterized since it has been thoroughly studied in petunia, snapdragon and maize [6]. Expression, functional and association studies have shown expression of structural genes in the anthocyanin biosynthetic pathway to be affected by the Myb and Myc family genes which also interact with each other [7]–[19]. Myb family genes have been observed to play an important role in the response to environmental factors, such as temperature and light [15], [20]–[22].

A number of genes in the biosynthetic pathway of anthocyanins have already been mapped to five different linkage groups (LG) and Myb transcription factors to two LGs [23]. Berry skin colour (with berries having non-coloured skin versus coloured skin) has been shown to have Mendelian segregation [23], [24]. This trait was mapped to LG2 and Salmaso *et al.* [23] mapped one transcription factor (*MybA1*) to the same locus [24]–[26]. Fournier-Level *et al.* [27] mapped colour as a quantitative trait to LG2.

Several studies have contributed to the better understanding on the regulation of anthocyanins by the Myb family genes. The absence of anthocyanins (i.e. white grapes) has been shown to be determined by the homozygous presence of a *MybA1* allele with a retrotransposon insertion (*Gret1*) in the gene promoter region [12]–[14], [19], [27]. *MybA1* and *MybA2* multiallelic mutations control the biosynthetic step mediated by *UFGT* [12], [28], [29]. However, some white cultivars do not have *Gret1* insertion and so it seems that this phenotype is also influenced by other genes [19]. Other transcription factors, *Myb5a*, *Myb5b*, *MybPA1* and *MybPA2*, have been found to affect expression of genes encoding enzymes involved in earlier steps of the pathway by the activation of the promoter [8], [10], [11], [15], [18]. However, none of these factors seem to play a role in regulating the UDP-glucose: flavonoids 3-*O*-glucosyltransferase (*UFGT*) [11].

Variation among cultivars with coloured berries has not been completely understood yet. Using a sample of 123 cultivars, This *et al.* [19] identified four polymorphisms in *MybA1* associated with pink/red cultivars. However, this study did not consider fruit colour quantitatively but instead defined it according to a 6-point scale. Recently, Fournier-Level *et al.* [27] used a sample of 141 cultivars to measure total skin anthocyanin (TSA) concentration in berry skin by high-performance liquid chromatography (HPLC) and identified four polymorphisms within *MybA1*, *MybA2* and *MybA3* genes that accounted for 23% of the variation. Anthocyanin content as a quantitative trait is expected to be determined by small contributions of many genes. In this study it was aimed to identify the remaining genetic contributions to the berry colour trait.

Although linkage mapping has been successfully used to identify major genes [30], [31] and QTL regions [32], [33], association mapping can provide higher power and resolution for the identification of variants. Association mapping studies may include the whole genome or candidate genes regions. In grapevine the latter is currently the only alternative since there are no genome-wide SNP arrays due to limited SNP discovery.

Power to detect genetic associations is influenced by sample size, linkage disequilibrium (LD) between the genotyped marker and the causal variant, effect size, and marker and causal variant frequencies. Several factors such as population structure, relatedness, poor study design and inaccurate phenotypic data can lead to spurious association [34]. However, population stratification and more recently cryptic relatedness have received a great deal of attention.

In grapevine and many other agricultural species, a certain degree of relatedness is expected due to selection and breeding history [35]. Yu *et al.* [36] developed a mixed model approach to account for population structure and cryptic relatedness while testing for genetic association. The model considers structure using the method presented by Pritchard *et al.* [37]. This method is based on a Bayesian clustering approach that estimates the proportion of each individual's variation that came from each subpopulation. This proportion is then included in the mixed model as a covariate. To account for relatedness, Yu *et al.* [36] used a model that includes a relatedness matrix based on a Ritland's kinship coefficient (RKC) [38]. This is estimated based on the probability of Identity by State (IBS) between two individuals adjusted to the average probability of IBS between random individuals in the population [36], [38]. Analyses based on this matrix can often lead to mathematical problems due to non-positive definiteness. However, Zhao *et al.* [39] suggested an alternative relationship matrix based on the proportion of shared alleles (PSA), a similarity measure first proposed by Chakraborty and Jin [40]. Zhao *et al.* [39] used haplotype data on 95 *Arabidopsis* accessions and showed with simulated data that this matrix is at least equally effective for taking into account relatedness. Using the matrix based on the PSA, Kang *et al.* [41] showed that this evades convergence and mathematical problems compared to the relationship matrix based RKC.

Despite the large number of studies on grape colour, there is still no clear understanding on the genetics underlying this phenotype. The studies performed to date, have focused on either presence or absence of colour, categorical variation of colour or total concentration of anthocyanins. This is the first study that examines a wide range of phenotypes including different types of anthocyanin concentration and relative abundance (RA). Obtaining samples for association studies in grapevine is still very challenging. This is because germplasm collections have often limited numbers of cultivars or cultivars with no phenotypic records. This study uses one of the largest samples for association mapping and sequence data to identify polymorphisms in grapevine. The aim is to look for associations between 15 candidate genes and grape colour using 124 newly discovered SNPs and a wide range of colour related phenotypes, including visual assessment, TSA concentration and specific types of anthocyanins concentration and RA. We also investigate the importance of population structure and relatedness in grapevine.

## Results

### Selection of markers

A total of 445 DNA polymorphisms, including 407 SNPs and 38 INDELs were identified within 15 candidate genes in 22 cultivars. Biological functions and expression analysis were the basis of gene selection. This list included genes encoding enzymes involved in the biosynthetic pathway of anthocyanins, in the transport of anthocyanins to the vacuole and genes encoding transcription factors of the *Myb* and *Myc* families. Evidence of subtle differential expression in Aragonez cultivar clones with contrasting TSA concentration supported the choice of the genes *MYC_B_*, *MYB9*, *MYB11* and *MYBCC* (unpublished results).

For the subsequent association analyses, a total of 124 SNPs were genotyped on 149 cultivars. The selection of this subset of SNPs was based on various quality control measures. High missingness (>20%) and low MAF (minor allele frequency) (<2%) were avoided. Preference was given to SNPs causing amino acid substitutions. SNP positions were considered in order to obtain coverage of the whole gene maintaining short distances between markers (average 300 bp). Also for this reason, in few regions with low quality sequence, four SNPs were retrieved from the SNP database hosted by The Institute for Genomic Research (TIGR), currently J. Craig Venter Institute (JCVI). The list of the genes and the number of SNPs within each gene are given in Table 1.

**Table 1:** List of candidate genes and number of SNPs genotyped on each for association analysis.

Chr	Scaffold (VITIS 8X)	Genoscope gene ID^1^	Code	Coded protein name	Function	Number of SNPs genotyped for association analysis
Unknown	168	GSVIVT00006341001	*CHS_A_* *	Chalcone synthase family	Involved in anthocyanins biosynthetic pathway. Catalyzes the condensation of one molecule of 4-coumaroyl CoA and three molecules of malonyl-CoA into a naringenin chalcone.	5
14	9	GSVIVT00037967001	*CHS_C_* *	Chalcone synthase family		7
13	48	GSVIVT00029513001	*CHI* *	Chalcone isomerase	Involved in anthocyanins biosynthetic pathway. Catalyzes the isomerization of the naringenin chalcone into a naringenin flavanone.	3
4	83	GSVIVT00036784001	*F3H* *	Flavanone 3-hydroxylase family	Involved in anthocyanins biosynthetic pathway. Catalyzes the hydroxylation of naringenin flavanone to dihydrokaemphenol.	5
17	12	GSVIVT00016215001	*F3′H_B_* *	Flavonoid 3′-hydroxylase family	Involved in anthocyanins biosynthetic pathway. Catalyzes the hydroxylation of dihydrokaemphenol at the 3′ position of the B-ring.	3
18	1	GSVIVT00014584001	*DFR* *	Dihydroflavonol reductase	Involved in anthocyanins biosynthetic pathway. Catalyzes the reduction of the dihydroflavonols into leucoanthocyanidins.	12
2	112	GSVIVT00001063001	*LDOX* *	Leucoanthocyanidin dioxygenase	Involved in anthocyanins biosynthetic pathway. Catalyzes the conversion of leucoanthocyanidins into anthocyanidins.	3
16	10	GSVIVT00014047001	*UFGT* *	UDP-glucose:flavonoids 3-*O*-glucosyltransferase	Involved in anthocyanins biosynthetic pathway. Catalyzes the conversion of anthocyanidins into anthocyanins	19
9	7	1XM_002276176	*MRP* *	Multidrug resistance–associated protein	Involved in vacuolar accumulation of anthocyanins in maize. ATP-binding transporter which mediates the primary transport of anthocyanins across the tonoplast.	14
Unknown	30	GSVIVT00023496001	*GST* *	Glutathione *S*-transferase	Involved in vacuolar accumulation of anthocyanins in grapevine. Thought to bind anthocyanins through hydrofobic interactions and escort them to the tonoplast membrane.	3
Unknown	203	GSVIVT00008627001	*MYC_A_* *	β helix-loop-helix transcription factor family	DNA-binding protein families with transcription factor activity. Some members described to be involved in regulation of the flavonoid and anthocyanin metabolism in other plants and in grapevine.	6
2	11	GSVIVT00015763001	*MYC_B_* §	β helix-loop-helix transcription factor family		10
9	7	GSVIVT00034097001	*MYB11* §	Myb transcription factor family		18
4	83	GSVIVT00036753001	*MYB9* §	Myb transcription factor family		4
Unknown	342	1XM_002272552.1	*MYBCC* §	Myb transcription factor family		12

1 Genoscope gene IDs are according to the sequencing version 8× coverage. NCBI locus nomenclature is shown for *MYBCC* and *MRP* because Genoscope annotation was not available in these cases.

* Candidate genes selection based on literature review.

§ Candidate genes selection based on previous expression analysis (unpublished results). Codes used to designate candidate genes selected based on expression analysis were retrieved from UniProt database description.

Data on SSR markers scattered across 18 chromosomes was used to measure background structure in the population sample and pairwise relationships between cultivars. Using the method presented by Pritchard *et al.* [37] to measure structure, two subpopulations were estimated. However, there were some indications of absence of structure, such as a near symmetric proportion of samples assigned to each subpopulation. Relatedness measures, based on PSA and RKC, were highly correlated (0.72, *P*<0.0001) and revealed some degree of relatedness among the individuals in the sample. Relatedness based on PSA ranged from 0.08 to 0.80, while relatedness based on RKC ranged from −0.20 and 0.82.

### Phenotypes

TSA concentration was measured by reversed-phase high-performance liquid chromatography (RP-HPLC) and expressed in mg of anthocyanins per kilogram of berries. The same method was used to measure concentration and relative abundance of specific anthocyanin types. Pulp colour (PC) was a visual characterization of coloured versus non-coloured pulp of berries. Skin and pulp colour (SPC) was also a visual characterization of berry colour, concerning skin as well as pulp colour. This classification included three categories, one with rose and red skinned cultivars with non-coloured pulp, a second with grey, dark red violet and blue black skinned cultivars and non-coloured pulp, and a third with cultivars showing coloured pulp. Other two visual classifications were used, and may be found together with more detail on all phenotypes in Table 2 and in Materials and Methods. Results from TSA concentration, PC, and SPC will be presented here in detail, while the results from the remaining phenotypes will be presented in Supporting Tables.

**Table 2:** List of the phenotypes used for association analysis.

Phenotypes		Concentration (mg of anthocyanins per kg of berries)	Relative abundance (%)	Variable type
Anthocyanins	Delphinidin-3-monoglucoside	√	√	Q
	Cyanidin-3-monoglucoside	√	√	Q
	Petunidin-3-monoglucoside	√	√	Q
	Peonidin-3-monoglucoside	√	√	Q
	Malvidin-3-monoglucoside	√	√	Q
	Delphinidin-3-monoglucoside-acetate	√	√	Q
	Cyanidin-3-monoglucoside-acetate	√	√	Q
	Petunidin-3-monoglucoside-acetate	√	√	Q
	Peonidin-3-monoglucoside-acetate	√	√	Q
	Delphinidin-3-monoglucoside-*p*-coumarate	√	√	Q
	Malvidin-3-monoglucoside-acetate	√	√	Q
	Peonidin-3-monoglucoside-caffeoate	√	√	Q
	Cyanidin-3-monoglucoside-*p*-coumarate	√	√	Q
	Malvidin-3-monoglucoside-caffeoate	√	√	Q
	Petunidin-3-monoglucoside-*p*-coumarate	√	√	Q
	Cis-Malvidin-3-monoglucoside-*p*-coumarate	√	√	Q
	Peonidin-3-monoglucoside-*p*-coumarate	√	√	Q
	Malvidin-3-monoglucoside-*p*-coumarate	√	√	Q
Total skin anthocyanin (TSA)		√		Q
Sum of anthocyanin groups	Sum of delphinidin derivatives	√	√	Q
	Sum of cyanidin derivatives	√	√	Q
	Sum of petunidin derivatives	√	√	Q
	Sum of peonidin derivatives	√	√	Q
	Sum of malvidin derivatives	√	√	Q
	Sum of monoglucosides	√	√	Q
	Sum of acetate derivatives	√	√	Q
	Sum of coumarate derivatives	√	√	Q
	Sum of caffeoate derivatives	√	√	Q
Ratio	Sum of coumaryl/Sum acetyl		√	Q
	Sum trihydroxylated/Sum dihydroxylated		√	Q
Visual colour characterizations	Pulp colour (PC)			D
	(categories: non-coloured pulp/coloured pulp)			
	Skin colour OIV 225 (SC)			P
	(categories: rose skin/red skin/grey skin/dark red violet skin/blue black skin)			
	Skin and pulp colour (SPC)			P
	(categories: rose and red skin with non-coloured pulp/grey, dark red violet and blue black skin and non-coloured pulp/coloured pulp)			
	Skin and pulp colour (SPC′)			P
	(categories: rose skin and non-coloured pulp/red skin and non-coloured pulp/grey skin and non-coloured pulp/dark red violet skin and non-coloured pulp/blue black skin and non-coloured pulp/coloured pulp)			

The last column shows variable types, where Q, D and P mean quantitative, dichotomous and polychotomous, respectively.

### Selection of statistical models

Several models were tested and compared (Tables S1 and S2, see Materials and Methods). F-tests between Model A and a model with structure effects (Model D) were significant for 94% of the markers (*P*<0.05). To assess the effect of relatedness, likelihood ratio tests were performed between models that included relatedness effects (Models B and C with matrices based on PSA and RKC, respectively) and a model with only SNP and structure effects (Model D). These tests were found significant for 98% and 99% of the markers, for relatedness based on PSA and RKC, respectively (*P*<0.05). The two different measures of relatedness were compared by likelihood ratio tests. By comparing the covariance parameters we found that the two matrices were not different for all the SNPs (*P*<0.01). Model B was selected for further association analysis because it raised fewer problems with convergence and non-positive definiteness. Model A was also used for all traits and SNPs as it is the more parsimonious model. Nominal and empirical *P*-values under Model A were obtained for TSA concentration, SPC and PC. The latter were based on 10 000 permutations. Models including the maturity of the berries and viral infections were not significant for TSA concentration (*P*<0.01). Therefore, these variables were excluded from the association analyses.

### Association results

#### Genes encoding transcription factors (*MYB11*, *MYBCC*, *MYC_B_*, *MYB9*, *MYC_A_*)

**Figure 1:**
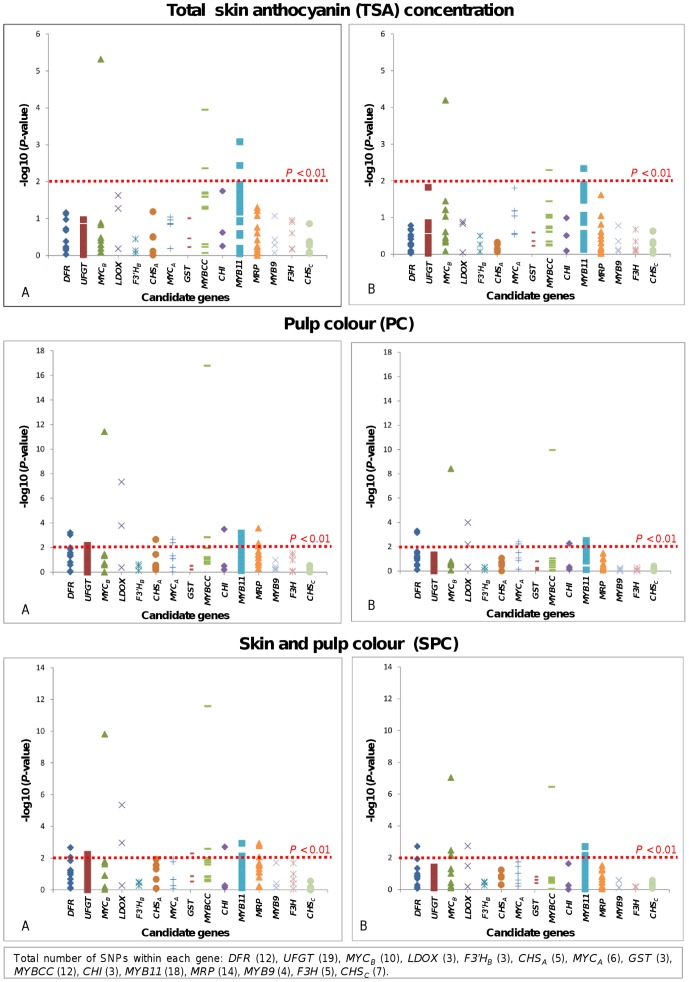
Results of association test of TSA concentration, PC and SPC. Graphs A and B show results for tests of association based on models A and B, respectively. The *y* axis shows −log10 (*P*-values). The different genes studied are shown along *x* axis. The total number of SNPs within each gene is indicated at the bottom.

Figure 1 shows the results of the associations using models A and B. The −log_10_ of the *P*-values were plotted on all genes for TSA concentration, PC and SPC. Table 3 shows the *P*-values for SNPs significantly associated with TSA concentration, SPC or PC for models A and B (*P*<0.01). *P*-values for all the tests performed under models A and B may be found on Tables S3 and S4.

**Table 3:** List of SNPs showing significant associations with total skin anthocyanin (TSA) concentration, pulp colour (PC) and skin and pulp colour together (SPC).

			TSA concentration		PC		SPC	
Gene	SNP ID	MAF	Model A	Model B	Model A	Model B	Model A	Model B
*MYB11*	s80	0.40			9.68×10^−03^			
	s83	0.38						9.34×10^−03^
	s84	0.49				3.11×10^−03^ *		2.04×10^−03^ *
	s86	0.40				5.14×10^−03^		7.11×10^−03^
	s87	0.07			2.95×10^−03^ *			
	s89	0.33	3.64×10^−03^		1.84×10^−03^ *		7.40×10^−03^	
	**s90**	**0.06**	8.29×10^−04^*	**4.64×10^−03^**	6.79×10^−04^*		1.14×10^−03^*	**9.73×10^−03^**
	s93	0.07			6.67×10^−03^			
	s94	0.35			5.43×10^−03^			
*MYBCC*	s65	0.21	4.31×10^−03^		1.47×10^−03^ *		9.61×10^−03^	
	**s68**	**0.06**	1.12×10^−04^*	**5.14×10^−03^**	1.64×10^−17^*	1.10×10^−10^*	2.62×10^−12^*	3.39×10^−07^*
	s71	0.48					2.55×10^−03^ *	
*MYC_B_*	s33	0.32						6.60×10^−03^
	s34	0.32						3.29×10^−03^ *
	**s36**	**0.15**	4.77×10^−06^*	6.29×10^−05^*	3.66×10^−12^*	3.66×10^−09^*	1.51×10^−10^*	8.84×10^−08^*
	s37	0.31						7.21×10^−03^
	s40	0.31						7.17×10^−03^
*MYC_A_*	s55	0.26			2.16×10^−03^ *	3.90×10^−03^		
	s58	0.27			4.00×10^−03^	5.88×10^−03^		
*CHS_A_*	s49	0.22			2.21×10^−03^ *			
*CHI*	s75	0.44			3.24×10^−04^ *	5.42×10^−03^	1.95×10^−03^ *	
*DFR*	s1	0.33			9.10×10^−04^ *	5.22×10^−04^ *	8.72×10^−03^	
	s11	0.03			6.18×10^−04^ *	7.41×10^−04^ *	2.14×10^−03^ *	1.86×10^−03^ *
*LDOX*	s42	0.19			4.63×10^−08^ *	1.49×10^−04^ *	4.41×10^−06^ *	1.80×10^−03^ *
	s44	0.28			1.65×10^−04^ *	6.33×10^−03^	1.07×10^−03^ *	
*UFGT*	s20	0.31			9.79×10^−03^			
	s22	0.32					9.94×10^−03^	
	s29	0.34					8.02×10^−03^	
	s30	0.37			6.78×10^−03^		5.77×10^−03^	
*MRP*	s95	0.26			2.64×10^−04^ *		1.42×10^−03^ *	
	s98	0.42			7.59×10^−03^		8.28×10^−03^	
	s100	0.30			6.85×10^−03^		1.62×10^−03^ *	
	s102	0.31			4.33×10^−03^		1.13×10^−03^ *	
*GST*	s59	0.15			8.02×10^−03^		4.99×10^−03^	

This table shows significant nominal *P*-values obtained under Models A and B (*P*<0.01). Significance was confirmed by 10 000 permutations for Model A. SNPs significant after Bonferroni correction for 15 genes are marked with *. Some SNPs that were not significant for TSA concentration, PC and SPC but were associated with other phenotypes are shown on Tables S3 and S4. Table S8 shows location, MAF, Hardy Weinberg chi-squared and missing data for all the SNPs genotyped.

Five SNPs (**s36**, s65, **s68**, s89, **s90**) across three different genes encoding transcription factors (*MYB11*, *MYBCC* and *MYC_B_*) yielded associations with TSA concentration using Model A (Table 3). Significance was also observed for **s36**, **s68** and **s90** with the mixed model for TSA (Model B). The SNPs **s36** and **s68** were also associated with SPC and PC under both models.

SNP s89 is synonymous and **s90** is in the predicted promoter region. For TSA and SPC, **s90** was significant for both models (Table 3). Three additional SNPs (s83, s84 and s86) were associated with SPC for Model B. SNPs s80, s87, s93 and s94 were associated with PC under Model A, and s84 and s86 for Model B.

Two intronic SNPs within *MYBCC* (s65 and **s68**) were associated with TSA concentration, PC and SPC (Table 3) under Model A. The SNP **s68** was significant for these 3 phenotypes with both models. The strongest associations were found between **s68** and PC using Model A and Model B. A third SNP (s71), also located on an intron region, was found to be associated with SPC for Model A. In *MYC_B_*, one synonymous SNP (**s36**) showed association with the three phenotypes under all the models. The highest significance was observed for PC with Model A. This SNP was also associated with a large percentage of phenotypes (33%; Table S5a). Four additional SNPs (s33, s34, s37 and s40) showed association with SPC under model B (Table 3). All the associations using Model A were verified empirically through permutations.

Overall, *MYB9 and MYC_A_* genes did not reveal any associations with TSA concentration and SPC. Two SNPs (s55 and s58), however, within *MYC_A_* showed association with PC under both models (Table 3).

For *MYB11*, *MYBCC* and *MYC_B_*, a high percentage of SNPs (approximately 80%) were found to be associated with at least one of the phenotypes for model A (*P*<0.01; Table S5a,b). In *MYB11*, over half of the phenotypes (53%) were associated with at least one of the SNPs under model A (*P*<0.01) (Table S5a,b). These phenotypes were mainly acetate and coumarate derivative anthocyanins (Table S3). In *MYBCC* many phenotypes (28%), mainly concentrations of different types of anthocyanins, were associated with at least one SNP under Model A (Table S5a,b). However, **s68** yielded the strongest signal (*P*<0.001; Table S6). Peonidin-3-monoglucoside concentration was associated with seven SNPs in this gene (58%) (*P*<0.01; Table S3). In *MYC_B_*, 44% of the phenotypes were associated with at least one of the SNPs (Table S5a,b).

Association between different phenotypes and the same gene are not surprising since all phenotypes are related to the colour of berries. However, correlation estimates between phenotypes showed a wide range of values from r^2^ = 0.83 (*P*<0.0001) between PC and SPC to r^2^ = 0.50 (*P*<0.0001) between TSA and these visual phenotypes.

Pairwise D′ was generally very high between SNPs within *MYB11*, *MYBCC* and *MYC_B_* (Table S7).

#### Genes encoding enzymes involved in the biosynthetic pathway of anthocyanins (*CHS_A_*, *CHS_C_*, *CHI*, *F3H*, *F3′H_B_*, *DFR*, *LDOX*, *UFGT*)

The SNP s30 on *UFGT* was associated with both PC and SPC under Model A (Table 3). Under this model two other SNPs (s22 and s29) were associated with SPC and one (s20) with PC. SNP s11 within *DFR* and in the 3′UTR region was associated with PC and SPC for both models. Another SNP (s1) in the predicted promoter region, was associated with PC and SPC using Model A. On *LDOX* the SNP s42 in the 3′UTR region associated under both models with PC and SPC showing the strongest significance for PC. The SNP s44 in the promoter region was associated with PC and SPC for Model A and with PC under Model B. The non-synonymous SNP s75 in *CHI* was associated with PC and SPC using Model A, and with PC for Model B.

No other genes encoding enzymes involved in the biosynthetic pathway of anthocyanins showed SNPs associated with TSA concentration or with SPC. *CHS_A_* showed one SNP (s49) to be associated with PC under model A (*P* = 2.21×10^−03^) (Table 3).

Overall, a high percentage of the SNPs (near or above 50%) within *UFGT*, *DFR*, *LDOX* and *CHI* showed association with at least one of the phenotypes under Model A (Table S5a,b). One third of the phenotypes within *UFGT* were associated with at least one of the SNPs using Model A (Table S5a,b). Interestingly, SNP s25, which causes an amino acid substitution, was the most important as it was associated with 25% of total phenotypes (Table S5a). Most phenotypes were included in the RA variable group, especially involving Peonidin derivatives (Table S6).

In general, these four genes showed great variation on MAF but LD was very high (Table S7 and S8). Values of D′ between the significant markers for TSA concentration, PC and SPC, were between 0.81 and 1 (Table S7).

#### Genes encoding enzymes involved in the transport and vacuolar accumulation of anthocyanins (*GST*, *MRP*)

Four SNPs within *MRP* gene (s95, s98, s100 and s102) were associated with PC and SPC under model A (Table 3). SNP s98 is synonymous, while the other three cause amino acid changes. For the *GST* gene, the intronic SNP (s59) was found to be associated with SPC and PC for Model A. All the SNPs within *GST* showed associations with at least one of the 61 phenotypes using Model A and 67% under Model B (S5a,b).

Using Model A, more than half of the SNPs (57%) within *MRP* genes were associated with the remaining phenotypes (Table S5a,b). Overall, these associations included 24 phenotypes (39%; Table S5a,b). Six of these polymorphisms were associated with a relatively high number of phenotypes, between six and 12 among the total of 61 phenotypes (Table S3). Concentration and RA of Peonidin derivatives were a large proportion of the associated phenotypes (Tables S6). The test using Model B showed a smaller number of SNPs (43%) and phenotypes (18%) to be associated (Table S5a,b) within *MRP* gene. This is a general trend to all studied genes with few exceptions (MYC_A_, CHS_C_, F3H).

Likewise to the remaining genes, LD across the *MRP* and *GST* was very high (Table S7).

Significant associations under Model A for the three main phenotypes (TSA, PC and SPC) were corrected for multiple testing using 10,000 permutations of the dataset. The association tests performed using the log transformed values of TSA concentration yielded similar results to models A and B (results not shown).

### Statistical interactions

Tests for statistical interactions were performed between SNPs within each of three transcription factors (*MYB11*, *MYBCC*, *MYC_B_*) and the remaining genes for TSA concentration. A number of interactions were observed between SNPs within several genes (*P*<0.001). *P*-values for these interactions are shown on Table S9. Only pairs of genes showing more than 25% of SNPs involved in significant interactions were further explored.

**Figure 2:**
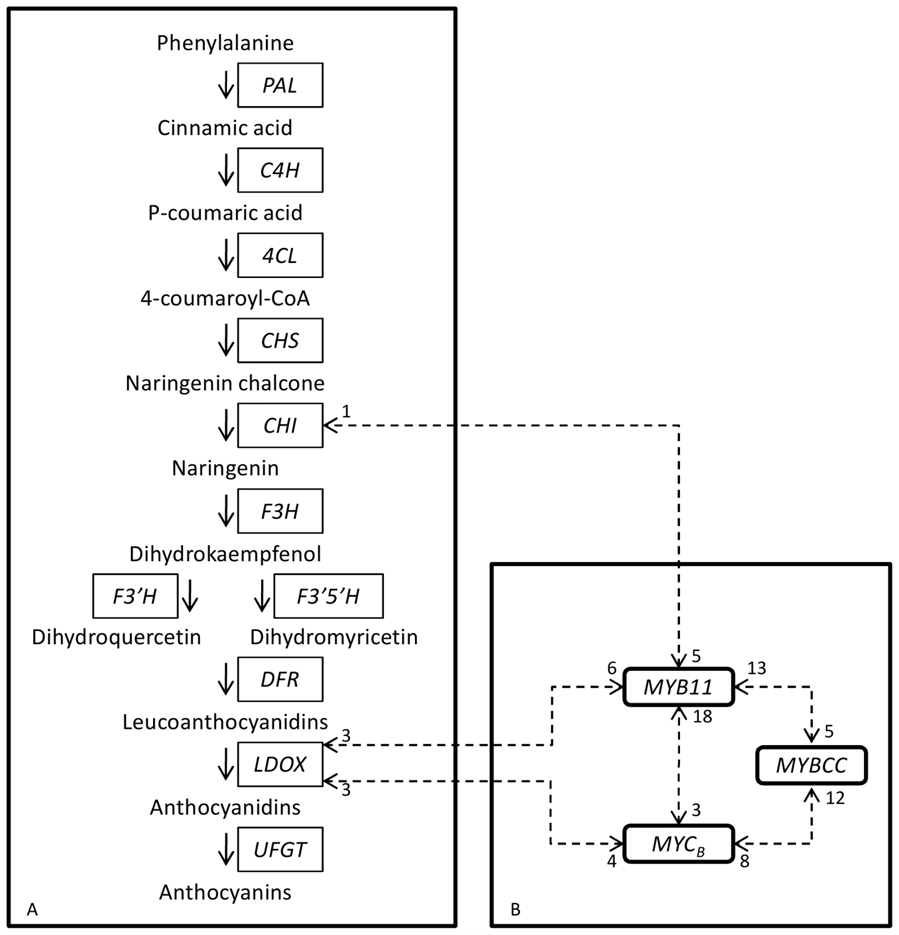
Schematic representation of the genes showing SNP×SNP statistical interactions. Scheme A shows a simplified biosynthetic pathway of anthocyanins. Scheme B shows the genes encoding transcription factors with SNPs (>25%) involved in SNP×SNP statistical interactions (*P*<0.001). The interactions are represented by dashed arrows and the numbers beside the arrows indicate the number of SNPs involved in significant interactions.

Figure 2 shows a schematic representation of the biosynthetic pathway of anthocyanins and of the pairs of genes within which SNP×SNP statistical interactions were observed. Scheme A shows a simplified biosynthetic pathway of anthocyanins. Scheme B shows the genes encoding transcription factors with SNPs (>25%) involved in SNP×SNP interactions (*P*<0.001). The statistical interactions are represented by dashed arrows and the numbers beside the arrows indicate the number of SNPs involved in significant SNP×SNP interactions.

Interactions between more than 25% of the SNPs were identified between *MYB11* and the following four genes: *LDOX*, *CHI*, *MYC_B_* and *MYBCC* (*P*<0.001; Table S10). This was also observed between the gene encoding *MYC_B_*, and *MYBCC* and *LDOX*. Table 4 presents the top six SNP×SNP interactions for which the model and the interaction effects were significant. These SNP×SNP interactions were within the *MYB11*, *MYBCC*, *MYC_B_*, *LDOX* and *CHI* genes. The results show that the significance of the model was as strong as or stronger than the single SNP tests. The significance values on Table 4 were confirmed by 1000 permutation tests.

**Table 4:** Interactions between SNPs in different genes.

Interactions	Model *P*-value	Interaction *P*-value	Single SNP tests *P*-values			
*MYB11*_s93_×LDOX_s42_	4.3×10^−04^	1.2×10^−03^	s93	4.4×10^−02^	s42	2.4×10^−02^
*MYB11*_s89_×*CHI*_s75_	2.4×10^−05^	2.6×10^−03^	s89	3.6×10^−03^	s75	1.8×10^−02^
*MYB11*_s90_×*MYC_B_*_s36_	1.2×10^−06^	1.6×10^−02^	s90	8.3×10^−04^	s36	4.8×10^−06^
*MYB11*_s91_×MYBCC_s65_	5.0×10^−05^	1.0×10^−03^	s91	3.4×10^−02^	s65	4.3×10^−03^
*MYC_B_*_s36_×MYBCC_s63_	4.9×10^−07^	4.3×10^−03^	s36	4.8×10^−06^	s63	2.5×10^−02^
*MYC_Bs_*_36_×*LDOX_s42_*	4.5×10^−06^	1.6×10^−02^	s36	4.8×10^−06^	s42	2.4×10^−02^

The first column shows the name of the two genes and in subscript the name of the SNPs in the model. The last column shows the *P*-values of the singles SNP tests under Model A. Both tests were performed with the phenotype TSA concentration. The model *P*-values were confirmed by 1000 permutation tests.

## Discussion

Population structure and more recently cryptic relatedness have been suggested as potential causes for spurious results in association studies. Here we examined structure and relatedness using two different matrices (PSA and RKC). We found that for more than 90% of the SNPs, the simplest model was significantly different to the full model that considered both effects. However, the analyses of the main phenotype (TSA concentration) showed that the simple and full model yielded similar results. These associations with the simple model were examined both nominally and empirically. So the question that arises is whether the use of a less parsimonious model could lead to type II errors. The statistical analysis showed also that the two relatedness matrices (based on PSA and on RKC) were not different which is in agreement with the simulation study performed by Zhao [39].

Three genes encoding transcription factors, *MYB11*, *MYBCC* and *MYC_B_* were found to be associated with TSA concentration. Three SNPs (**s36**, **s68**, **s90**) were associated with TSA concentration after correcting for relatedness and structure. Empirical *P*-values for these SNPs confirmed their association with TSA concentration. Moreover, SNPs **s36** and **s68** were also associated with SPC and PC under both models. None of the changes caused by these three SNPs are non-synonymous. SNP **s36** leads to a G/C base replacement causing no amino acid substitution. However, this sequence region is a CG and CHG context (where H = A, T or C), possibly important for epigenetic regulation by cytosine methylation [42]. Also there is a 12 bp INDEL 239.5 bp upstream of **s36** in the 5′UTR region. UTRs have been shown to play an important role on gene expression regulation. This influence may rely on different mechanisms such as the presence of upstream ORFs, secondary structures and protein or short RNAs binding sites [43]. SNP **s68** causes an A/C base substitution in an intron region. As this is a CHH sequence, it may also play a role in methylation [42]. It is now evident that intronic regions play important functional roles such as gene expression enhancement, alternative splicing and generation of regulatory RNA [43]. SNP **s90** causes an A/G base replacement in a sequence predicted to be part of the promoter region. According to the TSSP promoter prediction program for plant genes available on SoftBerry network server (http://www.softberry.com), **s90** is located only 4 bp upstream of the transcription start site (TSS). Two additional SNPs, s65 and s89, in *MYBCC* and *MYB11* respectively, were associated with TSA concentration under Model A. SNP s65 is located on an intron region and while s89 causes a mutation on an exon, it does not actually lead to an amino acid change. SNP s65 may play a regulatory role or change methylation on these regions since this is a CG or CHH sequence depending on the SNP allele. SNP s89 does not affect methylation [42] however 197.5 bp upstream of it is an 8 bp INDEL in the 5′UTR region, only 9 bp downstream of the predicted TSS. Empirical *P*-values confirmed the significance of these SNPs under the simple model.

Genes encoding *UFGT* and *MRP* were not associated with TSA concentration but were associated with individual anthocyanins, especially Peonidin derivatives and with phenotypes that were visually classified (PC and SPC). This indicates that these genes may be important for relative abundance of anthocyanins and less relevant for anthocyanin concentration. This is supported by the highest proportion of associated phenotypes involving relative abundance, especially in the case of *UFGT*. Visual characterizations of skin and pulp colour (PC and SPC) showed associations with a large number of genes, including genes coding transcription factors and related to the biosynthetic pathway and transport of anthocyanins.

The results showed variation on association between different phenotypes and the same SNP. Although all phenotypes are related to the colour of berries, correlation estimates between phenotypes varied substantially (Table S11). For example, the correlation between TSA concentration and the two visual phenotypes (PC and SPC) were found to be around 0.50 for both (*P*<0.0001). These two visual phenotypes were highly correlated (r^2^ = 0.83, *P*<0.0001) and so the association results were very similar. However, other factors could also be important. Different degrees of penetrance, phenotypic heterogeneity and environmental factors could all lead to differences in association. Also, there was variation between different SNPs for the same phenotype. The different levels of association could be due to differences in MAF for the causal genetic variant and the set of markers.

Statistical interactions between a high proportion of SNPs were observed for genes encoding transcription factors and genes encoding enzymes involved in the biosynthetic pathway of anthocyanins. SNPs within *MYB11* showed interactions with SNPs on genes encoding *LDOX*, *CHI*, *MYBCC* and *MYC_B_*. Also SNPs on *MYC_B_* showed interactions with SNPs on *MYBCC* and *LDOX*. These results show interactions either between genes encoding transcription factors or between these and genes involved in the anthocyanin biosynthesis pathway suggesting a regulatory role of the transcription factors over the pathway enzymes. Biological interpretation of the statistical interactions must be performed carefully and ideally must be supported by further investigation [44]. The observed statistical interactions suggest that the transcription factor *MYB11* has a regulatory role over the genes *CHI* and *LDOX*. Also *MYC_B_* is suggested to regulate *LDOX*. These results indicate that the three transcription factors *MYB11*, *MYBCC* and *MYC_B_* may functionally interact to regulate anthocyanin synthesis. This interaction may occur directly between these transcription factors or arise by means of other factors that have not been studied in the current investigation. This agrees with previous findings where transcription factors from Myb and Myc family were shown to regulate genes encoding anthocyanins biosynthetic enzymes in maize, petunia, *Arabidopsis* and grapevine [7]–[19]. In grapevine, different Myb genes, *MYBPA1* [8], [18], *MYBPA2* [18], *MYB5a* [15], *MYB5b* [11] and *MYBA1* [9], were found to activate *LDOX* promoter. Deluc *et al.* [10], [11] also showed the regulation of *CHI* by the transcription factors *MYB5a* and *MYB5b*. Members of Myb and Myc transcription factor families have also been previously shown to interact with each other in grapevine and in other species. Differential expression analysis and transient expression assays showed that the interaction between the different transcription factors was essential for their ability to activate pathway genes expression [7], [8], [17], [45].

This study was performed on a small population sample compared to human genetics studies. However, in the area of genome research in grapevine, this is one of the largest samples studied for association mapping. Power calculations are based on the assumption of strong LD between the variant and the marker. Here we have performed fine mapping with average distance between SNPs of near 300 bp which makes the study powerful. Concerning multiple testing, Bonferroni correction is highly conservative as all the markers within the genes are in strong LD. Therefore, replication studies would be valuable for verifying the significance of these results. Despite the rapid increase in genomic resources for *Vitis vinifera* L., these are still limited compared to other species such as human. Association mapping is much more recent in plant studies. The availability of larger collections with genomic and phenotypic data would greatly contribute to future association studies. The International HapMap Project has been extremely useful and successful and such large scale studies will soon be available for other species including *Vitis*. The novel findings from this study and the SNPs that have been identified will be of great interest in genome-wide projects. This study has shown association between berry colour and anthocyanin content with interesting genes which need to be further investigated to better understand the genetics underlying colour. The identification of the functional variants will accelerate grapevine breeding programs.

## Materials and Methods

### Candidate genes

This candidate gene study was divided in two phases. Phase I involved SNP discovery by sequencing 22 cultivars. Phase II involved association analyses by genotyping 124 SNPs in 149 cultivars. The selection of genes was based on their biological functions and expression analysis. Table 1 shows the list of selected genes for this study and the source of information supporting their selection. The name and symbol of the encoded proteins are listed as well as the number of SNPs genotyped in each gene. A total of 15 candidate genes were selected, including genes encoding enzymes which are involved in the biosynthetic pathway of anthocyanins, to the transport of anthocyanins to the vacuole and genes encoding transcription factors of the Myb and Myc families. The genes *MYC_B_*, *MYB9*, *MYB11* and *MYBCC* showed subtle differential expression in Aragonez cultivar clones with contrasting TSA concentration (unpublished results).

### PCR and sequencing of 22 cultivars

Cultivars with coloured and non-coloured pulp and with a range of skin colours were included. Approximately 100 mg of leaf fresh weight was used to extract genomic DNA. Mortar and pestle grinding with sterile quartz sand were used with Quiagen Mini Kit (Quiagen Inc, Hilden, Germany). Sequences available on NCBI were used to design the primers with Primer3 software [46]. Primers were designed to amplify DNA fragments covering the candidate genes and known or predicted promoter regions. Published characterization of promoters was available for *CHI* [8], *DFR* [47], *UFGT* [48] and *LDOX* [49]. In the cases where promoters were not described, the TSSP promoter prediction program for plant genes available on SoftBerry network server (http://www.softberry.com) was used to predict the transcription start site and to identify promoter motifs up to 2000 bp upstream of the start site. PCR assays were performed by standard methods. Automated sequencing was performed by STAB Vida, Lda. (Portugal). SNPs were identified using CodonCode Aligner software (Codon Code Corp.).

### SNP selection and genotyping

A total of 445 DNA polymorphisms, including 407 SNPs and 38 INDELs, were identified. The selection of SNPs for further genotyping was based on various quality control measures. To avoid reducing power and including genotyping errors, SNPs with MAF <2% were excluded. SNPs with missingness >20% were also avoided, as a stricter threshold would greatly reduce the sample. SNPs causing amino acid substitutions were preferred but the whole gene was covered with an average distance between SNPs of 300 bp. More than three SNPs within 20 base pairs intervals were avoided due to genotyping technology restrictions. For the few regions with low quality sequence, four SNPs were retrieved from the SNP database hosted by The Institute for Genomic Research (TIGR) in order to ensure good gene coverage (Table S8). Following these selection criteria 140 SNPs in total were selected across the 15 genes for genotyping in a larger sample of 149 cultivars. These cultivars with coloured berries were collected on the same vineyard in Dois Portos, Portugal, where the national ampelographic collection is established. SNP genotyping was performed using KasPar technology at KBiosciences (Hertfordshire, UK). Quality control analyses were performed. A total of 124 SNPs were obtained after filtering for MAF <0.02, HW (Hardy-Weinberg) deviations with χ^2^>10 and with missingness >20% (Table 1). The genotyped SNPs were on average near 300 bp apart allowing very fine mapping (Table S8). Measures of pairwise LD were obtained showing strong LD across all genes. These values are presented in Table S7.

### Phenotypes

The phenotypes included a wide range of traits based on either RP-HPLC analysis or visual characterization. Phenotypes based on RP-HPLC analysis included TSA concentration but also specific types of anthocyanins and relative abundance (RA). Every anthocyanin was grouped based on the type it belonged to, for example anthocyanidin and acylation types. Additionally, ratios between di/trihydroxylated anthocyanins and coumarate/acetate derivatives were used as phenotypes. The entire list of phenotypes is presented in Table 2.

Probable alcohol percentage was used as an indicator of berry maturity state at harvest. A total of 50 berries divided by two replicates were collected from each cultivar with approximately 9% probable alcohol. Extraction of anthocyanins from berry skin was performed using acidified methanol. The extracts were analyzed using RP-HPLC. Photodiode array spectra were recorded between 250 and 650 nm and chromatograms were acquired at 525 nm. Individual identification of anthocyanins was based on retention times and absorption spectral properties. Concentrations were calculated using a calibration curve obtained by regression through the origin of RP-HPLC peak areas on concentration (in mg/l) of an external pattern of malvidin-3-O-glucoside chloride (Hoffman-La Roche, Switzerland).

Pulp colour (PC) is a dichotomous trait (coloured versus non-coloured pulp). Skin colour (SC) was classified according to descriptor number 225 by the International Organization of Vine and Wine (OIV). This descriptor establishes the following five categories: rose, red, grey, dark red violet and blue black. OIV descriptor 225 has a certain degree of subjectivity and PC is likely to influence to some extent the classification of SC. Since these visual phenotypes were aimed to be used for genetic association analysis, two new classifications were tested targeting higher accuracy. These classifications were named SPC and SPC′ and were obtained by joining pulp and skin colour classifications (Table 2). Only three categories for SPC were established. The first included cultivars with rose and red skin berries with non-coloured pulp. The second included grey, dark red violet and blue black skin cultivars with non-coloured pulp and the third included only coloured pulp cultivars. For SPC′ a sixth category for coloured pulp cultivars was added to the five OIV225 categories (Table 2).

Other variables which could interfere with anthocyanin content of the berries were measured. Traits related with the maturity of berries were measured by the Central Laboratory of the Instituto Nacional de Investigação Agrária (INIA-Dois Portos) during the anthocyanin extraction. Brix degree (% m/m), sugar content (g/l), volumic mass (g/cm3) and probable alcohol (% v/v) were determined by refractometry. Total acidity (g/l tartaric acid) was measured by colorimetric titration. All these measurements were performed according to OIV method [50]. Several viral infections were assessed with the ELISA test. These were grapevine virus B (GVB), grapevine fanleaf (GFLV), arabic mosaic (ArMV), grapevine fleck (GFKL) and grapevine leafroll-associated viruses (GLRaV1, GLRaV2, GLRaV3, GLRaV7). These tests were performed by the National Institute of Biologic Resources. None of these covariates were found significant when tested for TSA concentration using a stepwise regression (*P*<0.01). Therefore, these covariates were excluded from the association analyses. All statistical analyses were carried out using SAS v9.1 (SAS Institute Inc., Carry, NC, USA).

### Structure

Data on 20 SSR loci scattered across 18 different chromosomes for 149 cultivars were provided by the Istituto Agrario San Michele all'Adige (IASMA). These loci were independent and had a high Polymorphic Information Content (PIC), with an average of 0.7. These 20 SSR loci were used to assess background structure in the population sample. The program STRUCTURE [37] was used to obtain an estimate of the number of subpopulations (*K*). It was assumed that each individual drew some fraction of its genome from each of the *K* populations and that allele frequencies in these populations were correlated. The parameter α which was used to model the degree of admixture was inferred from the data [37]. Lambda, the parameter of the distribution of allele frequencies, was set to the default value of 1. The Markov chain Monte Carlo was performed with a burn-in period of 500 000 iterations followed by 500 000 iterations. According to pilot runs, summary statistics (alpha, divergence distances among populations and joint probability) were stable for this length and did not increase for a length of 1 000 000 iterations. Moreover, estimates of joint probabilities, allele frequencies and proportion of admixture were consistent between different runs for the same number of subpopulations (*K*). The presence of two subpopulations was suggested since *K* = 2 was the smallest among several values of *K* giving similar estimates of log probabilities. However, alpha showed a high range towards the end of iterations and the proportion of samples assigned to each subpopulation was nearly the same, suggesting the absence of structure [51]. Data on the same runs were also used to assess the number of subpopulations according to Evanno *et al.* [52]. This method also gave *K* = 2; however, it is not appropriate to detect the absence of structure since it cannot find the optimal *K* if *K* = 1.

### Relatedness

The same data (20 SSR loci) were used to measure pairwise relationships using two different methodologies. Firstly, a pairwise relationship based on the PSA between pairs of individuals was calculated as proposed by Chakraborty and Jin [40]. The second measure of pairwise relationship was based on Ritlands' kinship coefficient (RKC) using the SPaGeDi software [38], [53].

The relatedness matrix based on RKC was transformed prior to any analysis. Negative values were set to zero, as this means that they are less related than a random pair of individuals [53]. Diagonals were equal to 1+F, where F was the inbreeding coefficient obtained by SPAGeDi [54]–[56]. All the off-diagonals were transformed to pairwise relationships between cultivars by multiplying by two the kinship coefficient [54], [55]. The matrix of PSA was used in the mixed model without any alterations. Both methods, revealed some degree of relatedness among the individuals in the sample. Correlation between the two pairwise estimates was high (0.72, *P*<0.0001) and was confirmed by permutation analysis [57]. Relatedness based on RKC ranged from −0.20 to 0.82, while relatedness based on the PSA ranged from 0.08 to 0.80.

### Association models

Several models were applied and compared. A list of the different models used is shown on Table S1 and a list of the comparisons performed on Table S2. Model comparisons were performed using the phenotype of TSA concentration. The selected models were used to test association for the remaining phenotypes.

The simplest model tested, Model A, was a linear regression with phenotype as the response variable and SNP as the predictor variable. The genotypes AA, Aa, aa were coded as 0, 1, and 2, respectively. Model B was as Model A but it included the relationship matrix based on PSA as a random effect and structure as a covariate. Model C was identical to Model B, but RKC matrix substituted PSA matrix. Model D was similar to Model A but with structure added. Finally, models E and F were similar to models B and C but excluding structure (Table S1). The SAS PROC MIXED procedure was used for all the mixed model analyses.

To assess the importance of structure in the association analyses, two models were compared by F-test (comparison 1; Table S2). The percentage of markers for which Model D results were different from Model A was above 90% (*P*<0.05) and therefore it was decided to include structure in further analysis. To assess the importance of relatedness in the association model, likelihood ratio tests were performed (comparisons 2a and 2b; Table S2). In these comparisons, the reduced model was Model D and the full models were models B and C. These comparisons revealed differences (*P*<0.05) for more than 90% of the markers. As the percentage of markers for which the models B and C were different from Model D was very high, relatedness was included in further analyses.

To assess the significance of the two different measures of relatedness, a likelihood ratio test was used (comparison 3; Table S2). Comparing the covariance parameters for Models E and F we found that the two matrices were not different for all markers (*P*<0.01). Therefore, relatedness based on PSA was used since it raises fewer problems with convergence and non-positive definiteness. Tests of association were performed with two different models, Model A and Model B. All statistical analyses were performed with the original phenotypes and the log transformed values but the results were essentially the same. Throughout the study we present only the analyses performed with the original phenotypes. Also, correction for multiple testing was performed by 10 000 permutations for TSA concentration, PC and SPC under Model A.

### Statistical interactions

Gene-gene interactions were tested using SNP data on a model for two-locus interactions. Statistically, these tests were performed by using a multiple regression model where the phenotype was regressed on genotype on locus 1, genotype on locus 2 and on interaction between loci 1 and 2, according to the following formula:
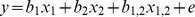
On this multiple regression model, *y* represents phenotypic observations; *x_1_* is SNP1 genotypes; *b_1_* is the regression coefficient for *x_1_*; *x_2_* is SNP2 genotypes; *b_2_* is the regression coefficient for *x_2_*; *x_12_* is interaction between the two loci; and *b_12_* is the regression coefficient for *x_12_*.

Interaction tests were performed for TSA concentration between each of the three transcription factors (*MYB11*, *MYBCC*, *MYC_B_*) and the remaining genes. These three transcription factors were selected for interactions due to the associations shown with TSA concentration on single SNP tests. Transcription factors were also especially interesting since previous works have shown interactions among different transcription factors, between these and genes involved in the biosynthetic pathway of anthocyanins and also between transcription factors and genes related to anthocyanin transport [8], [9], [15], [18], [27]. Results for significant interactions are shown on Tables S9 and S10. As SNPs within each gene, phenotypes and models are strongly correlated, correction for multiple testing was performed by 1000 permutations of the dataset to the top six SNP×SNP interactions for which the model and the interaction effects were significant.

## Supporting Information

Table S1 **List of the statistical models tested.**
(DOC)

Table S2 **List of model comparisons performed.**
(DOC)

Table S3 ***P*** **-values for all the association tests carried under Model A.**
(XLS)

Table S4 ***P*** **-values for all the association tests carried under Model B.**
(XLS)

Table S5 **a.** Percentage of phenotypes showing significant associations (*P*<0.01) with each SNP. **b.** Percentage of SNPs and phenotypes showing significant associations (*P*<0.01) for each gene.
(XLS)

Table S6 **Percentage of variable groups associated with each gene under Model A.**
(DOC)

Table S7 **Pairwise LD values estimated between SNPs within each gene.**
(XLS)

Table S8 **Location, MAF, Hardy Weinberg chi-squared and missing data for all the SNPs genotyped.**
(XLS)

Table S9 **Model and interaction** ***P*** **-values of significant interactions between SNPs in different genes (** ***P*** **<0.001).**
(XLS)

Table S10 **Percentage of SNPs involved in significant interactions.**
(XLS)

Table S11 **Correlation matrix between visual assessment of berry colour, relative abundance and concentration (mg/kg) of anthocyanins.**
(XLS)
