# Supplementary material for: A Candidate-Gene Association Study for Berry Colour and Anthocyanin Content in Vitis vinifera L
Source: PLoS One. 2012 Sep 28;7(9):e46021. doi: 10.1371/journal.pone.0046021 (PMC3461038; doi:10.1371/journal.pone.0046021)
Supplement: Table S1 — List of the statistical models tested. (DOC) [file pone.0046021.s001.doc]

Supporting Table S1. List of the statistical models tested.

| **Model name** | **Model in matrix notation** | **Description** | |
| --- | --- | --- | --- |
| A | $y=X\beta+e$ | *PHE = SNP* | Regression model where phenotype is the response variable and genotype (0, 1, 2) is the independent variable. |
| B | $y=X\beta+Q\gamma+Z\delta+e$ | *PHE = SNP + Q + PASR* | Same as Model A, but with structure and relatedness based on PSA included. |
| C | $y=X\beta+Q\gamma+Z'\delta'+e$ | *PHE = SNP + Q + RKCR* | Same as Model A, but with structure and relatedness based on RKC included. |
| D | $y=X\beta+Q\gamma+e$ | *PHE = SNP + Q* | Same as Model A, but with structure included. |
| E | $y=X\beta+Z\delta+e$ | *PHE = SNP + PASR* | Same as Model A, but with relatedness based on PSA included. |
| F | $y=X\beta+Z'\delta'+e$ | *PHE = SNP + RKCR* | Same as Model A, but with relatedness based on RKC included. |

In the formula *y* is a vector of phenotypic observations (TSA concentration), *β* is a vector of SNP allele effects to be estimated, *X* contains the genotypes*, γ* is a vector of population structure effects, Q is a matrix with the proportion of individuals genome inherited from ancestors in each subpopulation inferred by STRUCTURE*, δ* is a vector of random effects due to relatedness based on PAS, *Z* is an incidence matrix relative to *δ, δ’* is a vector of random effects due to relatedness based on RKC, *Z’* is an incidence matrix relative to *δ’,* and  *e* is a vector of residual effects.

In the schematic representation *PHE* means phenotypic data (TA concentration), *SNP* means genotype data on SNPs, *Q* means population structure measured as the proportion of individuals genome inherited from ancestors in each subpopulation, *PASR* represents pairwise relationships between individuals based on the PSA and *RKCR* represents the pairwise relationships between individuals based on RKC.
