# Supplementary material for: A Candidate-Gene Association Study for Berry Colour and Anthocyanin Content in Vitis vinifera L
Source: PLoS One. 2012 Sep 28;7(9):e46021. doi: 10.1371/journal.pone.0046021 (PMC3461038; doi:10.1371/journal.pone.0046021)
Supplement: Table S2 — List of model comparisons performed. (DOC) [file pone.0046021.s002.doc]

Supporting Table S2. List of model comparisons performed.

| **Model comparison** | **Test** | **Reduced model** | **Full model** | **Objective** |
| --- | --- | --- | --- | --- |
| 1 | F-test | A *(PHE = SNP)* | D (*PHE = SNP + Q)* | Assess importance of structure in the association model. |
| 2a | Likelihood Ratio Test | D (*PHE = SNP + Q)* | B (*PHE = SNP + Q + PASR)* | Assess importance of relatedness using PSA in the association model. |
| 2b | Likelihood Ratio Test | D (*PHE = SNP + Q)* | C *(PHE = SNP + Q + RKCR)* | Assess importance of relatedness using RKC in the association model. |
| 3 | Likelihood Ratio Test | E (*PHE = SNP + PASR)* | E’ (*PHE = SNP + PASR)* | Assess differences in importance of relatedness in the association model using RKC or PSA. |

E’ stands for model E but with covariance parameters set using the covariance parameters estimated for model F (Table S1).
