# Supplementary material for: A Candidate-Gene Association Study for Berry Colour and Anthocyanin Content in Vitis vinifera L
Source: PLoS One. 2012 Sep 28;7(9):e46021. doi: 10.1371/journal.pone.0046021 (PMC3461038; doi:10.1371/journal.pone.0046021)
Supplement: Table S6 — Percentage of variable groups associated with each gene under Model A. (DOC) [file pone.0046021.s006.doc]

**Supporting Table S6. Percentage of variable groups associated with each gene under Model A.**

| **Candidate genes** | | | | | | | | | | | | | | | |
| --- | --- | --- | --- | --- | --- | --- | --- | --- | --- | --- | --- | --- | --- | --- | --- |
| **Variable groups*** | ***MYC_A_*** | ***MYC_B_*** | ***MYB9*** | ***MYB11*** | ***MYBCC*** | ***CHS_A_*** | **CHS_C_** | ***CHI*** | ***F3H*** | ***F3’H_B_*** | ***DFR*** | ***LDOX*** | ***UFGT*** | ***MRP*** | ***GST*** |
| Relative abundance | 67 | 62 | 100 | 31 | 14 | 50 | 100 | 30 | 75 | . | 46 | . | 65 | 49 | 50 |
| Concentration | . | 32 | . | 60 | 70 | 33 | . | 50 | 25 | . | 18 | 50 | 21 | 36 | . |
| Delphinidin derivatives | 33 | . | . | 8 | . | . | . | . | 25 | . | . | . | 15 | 21 | . |
| Cyanidin derivatives | . | 21 | . | 15 | 3 | . | 20 | . | . | . | . | . | . |  | . |
| Petunidin derivatives | 33 | 9 | . | 10 | . | . | 20 | . | . | . | . | . | 15 | 6 | . |
| Peonidin derivatives | . | 19 | . | 13 | 38 | 67 | . | 20 | 25 | . | 27 | 25 | 33 | 38 | . |
| Malvidin derivatives | . | 15 | . | 23 | 16 | 17 | 20 | 40 | 25 | . | 18 | 13 | 6 | 11 | 50 |
| Glucoside derivatives | 33 | 23 | . | 7 | 43 | 33 | 20 | 20 | 25 | . | 9 | 25 | 21 | 21 | . |
| Acetate derivatives | . | 13 | . | 39 | . | 17 | . | . | . | . | . | . | 27 | 8 | . |
| Coumarate derivatives | . | 34 | . | 39 | 14 | . | 80 | 30 | 25 | . | 36 | . | 9 | 28 | 50 |
| Caffeoate derivatives | . | 4 | . | . | 3 | . | . | 10 | . | . | . | . | . | 2 | . |
| Visual characterizations | 33 | 6 | . | 8 | 16 | 17 | . | 20 | . | . | 36 | 50 | 15 | 15 | 50 |
| Ratios | . | . | 100 | . | . | . | . | . | 25 | . | 9 | . | 12 | 6 | . |

^*^Each variable group represents only those phenotypes that are relative to concentration, RA, anthocyanidin type, acylation type and ratios. Values are rounded to the unit. Zero percent is denoted by a dot “.”.
